# Single-cell RNA sequencing revealed the liver heterogeneity between egg-laying duck and ceased-laying duck

**DOI:** 10.1186/s12864-022-09089-0

**Published:** 2022-12-28

**Authors:** Xue Du, Shujing Lai, Wanqiu Zhao, Xiaoqin Xu, Wenwu Xu, Tao Zeng, Yong Tian, Lizhi Lu

**Affiliations:** 1 State Key Laboratory for Managing Biotic and Chemical Threats to the Quality and Safety of Agro-Products, Institute of Animal Husbandry and Veterinary Medicine, Zhejiang Academy of Agricultural Sciences, Hangzhou, 310021 Zhejiang China; 2 College of Animal Science and Technology, College of Veterinary Medicine, Zhejiang A & F University, Hangzhou, China; 3 Shanghai Institute of Immunology, Shanghai Jiao Tong University School of Medicine, Shanghai, China; 4 Institute of Horticulture, Zhejiang Academy of Agricultural Sciences, Hangzhou, 310022 Zhejiang China; 5 Institute of Ecology, China West Normal University, Nanchong, 637002 Sichuan China

**Keywords:** Ceased-laying duck, Single-cell RNA-seq, Cell heterogeneity, Hepatocytes, Cell cluster

## Abstract

**Background:**

In the late phase of production, ducks untimely cease laying, leading to a lower feed conversion. Liver plays a vital role in the synthesis and transport of yolk materials during egg formation in birds. However, the molecular mechanism of liver in ceased-laying duck is far from clear, higher resolution and deeper analysis is needed. Sing-cell RNA-sequencing of *10* × *Genomics* platform can help to map the liver single cell gene expression atlas of Shaoxing duck and provide new insights into the liver between egg-laying and ceased-laying ducks.

**Results:**

About 20,000 single cells were profiled and 22 clusters were identified. All the clusters were identified as 6 cell types. The dominant cell type is hepatocyte, accounted for about 60% of all the cells. Of note, the heterogeneity of cells between egg-laying duck and ceased-laying duck mainly occurred in hepatocytes. Cells of cluster 3 and 12 were the unique hepatocyte states of egg-laying ducks, while cells of cluster 0 and 15 were the unique hepatocyte states of ceased-laying ducks. The expression mode of yolk precursor transporters, lipid metabolizing enzymes and fibrinogens were different in hepatocytes between egg-laying duck and ceased-laying duck. *APOV1*, *VTG2*, *VTG1*, *APOB*, *RBP*, *VTDB* and *SCD* might be activated in egg-laying ducks, while *APOA1*, *APOA4*, *APOC3*, *FGB* and *FGG* might be activated in ceased-laying ducks.

**Conclusions:**

Our study further proofs that *APOV1* and *APOB* play key roles in egg production, rather than *APOA1* and *APOA4*. It is also the first to detect a correlation between the higher expression of *APOC3*, *FGB*, *FGG* and ceased-laying in duck.

**Supplementary Information:**

The online version contains supplementary material available at 10.1186/s12864-022-09089-0.

## Background

Eggs are well received as an important source of nutrients all around the world, as they are rich in proteins, lipids, vitamins and minerals. In Asia, duck eggs are traditional ingredients in both fresh style and processed style. However, the per capita share of duck eggs is still low, which results from farming scale with short peak laying period. In the group, some ducks meet untimely ceased-laying at the egg producing period which leads to a rapid downward trajectory of laying rate after the peak production. In late phase, ducks are known to be characterized by the declined production performance compared with those at peak production, resulting in a restricted economic benefit of egg-laying duck industry.

As a great metabolic factory, liver supports the energy demands of yolk formation in avian species. Liver provides lipids, proteins, vitamins, and choline, and so on, which are essential materials for yolk precursor synthesis [1]. Some studies detected the change of lipid metabolism in liver between pre-laying and peak-laying of laying hens [2, 3]. In consequence, the laying performance of duck might be improved by affecting hepatic lipid metabolism. For instance, dietary betaine improves chicken egg-laying rate through hypomethylation and glucocorticoid receptor-mediated activation of lipogenesis related genes in liver [4]. Previously, we revealed lipid metabolic genes in liver relate to yolk ratio of duck [5]. Based on these studies, we speculated that liver plays heterogeneous functions in the late phase of lower production compared with peak production.

The balance between lipogenesis and export of very-low-density apolipoprotein (VLDL) particles in liver is extremely important for egg production [6]. Peroxisome proliferator activated receptor (PPAR) might play great role in liver metabolism in the switch from egg-laying period to ceased-laying period. The lipid metabolism-related genes such as fatty acid binding proteins (*FABPs*), lipoprotein lipase (*LPL*) and apolipoprotein A1 (*APOA1*) that mapped to PPAR signaling pathway were downregulated in the late phase of production of hens [7]. Hawthorn-leaves flavonoids could improve the reproduction performance of aged breeder hens (60-wk-old) through upregulating the mRNA expression of vitellogenin II (VTG2), Apolipoprotein B (APOB), and apolipoprotein-VLDL II (APOV1) in the liver [8].

APOB and APOV1 are major components of yolk oriented VLDL (VLDL-yolk), which are produced by liver and function to transport lipids to the follicles for yolk deposition. The expression levels of these apolipoproteins dramatically elevate in laying hens compared with non-laying hens [9]. In sexually mature hens, enzymes participate in lipids synthesis and transport are basically controlled by sex hormones from hypothalamus, pituitary and other glands [10]. In hypothalamus, deep-brain photoreceptors (DBPs) extend fibers to the external zone of the median eminence adjacent to the pituitary to translate light information into downstream neuroendocrine responses [11]. Expression of thyroid-stimulating hormone β subunit (TSHB), deiodinase 2 (DIO2), organic anion transporting polypeptides (OATPs) are subsequently enhanced, which modulate gonadotropin-releasing hormone (GnRH) secretion from the hypothalamus [12–14]. GnRH regulates the release of gonadotropins, such as luteinizing hormone (LH), follicle-stimulating hormone (FSH), estrogen and progesterone hormones which subsequently arouses egg laying [15].

The conventional RNA sequencing of bulk sample helps to systematically assess gene expression level and reveal molecular regulatory networks in biological processes. It shows limitation for low-abundance cells, which unable to reveal key messages from complex and highly heterogeneous organisms. High-throughput single-cell RNA sequencing (scRNA-seq) breaks the conventional bulk sample-based experimental object, can reveal genes expression state of individual cell and reflect the heterogeneity between cells. scRNA-seq has been widely applied in the studies of reproduction, immunization, tumorigenesis and differentiation. In recent years, the cell atlas of mouse and human have been published successively [16, 17], which illustrate the trajectory of cell development and gene expression dynamics in cell differentiation. In poultry science, using scRNA-seq, Li et al. [18] described the heterogeneity of chicken skeletal muscle at two developmental stages, and identified *APOA1* (apolipoprotein A1) and *COL1A1* (the type I collagen encoding gene) as biomarkers for chicken intramuscular fat cells. Shaoxing duck is a sexually mature early, high-yielding duck species. In this study, we constructed a single-cell resolution transcriptomic atlas of liver in laying and ceased laying ducks based on Shaoxing duck in the late phase of production. Our study might reveal the inner view of the mechanisms of untimely ceased-laying in duck liver, thereby providing potential targets for improving the performance of egg-laying ducks in the late phase of production.

## Results

### Sequences statistic and quality control

We obtained over 28.4 M reads in each sample. The sequence data showed a high quality, with valid barcodes over 96%, Q30 bases in barcode over 95%, Q30 bases in RNA read over 93% and Q30 bases in UMI over 92% (Supplementary Table S1). On the whole, over 86.6% of the reads matched to the reference, with an average of 538 and 640 genes detected per cell in L_C and L_L, respectively. Median number of genes detected per cell as a function of mean reads per cell was shown in Supplementary Figure S1. Over 29 k of mean reads were detected per cell, and over 16 k of the total genes were detected (Supplementary Table S2).

### Transcriptional clustering of cell populations

**Fig. 1:**
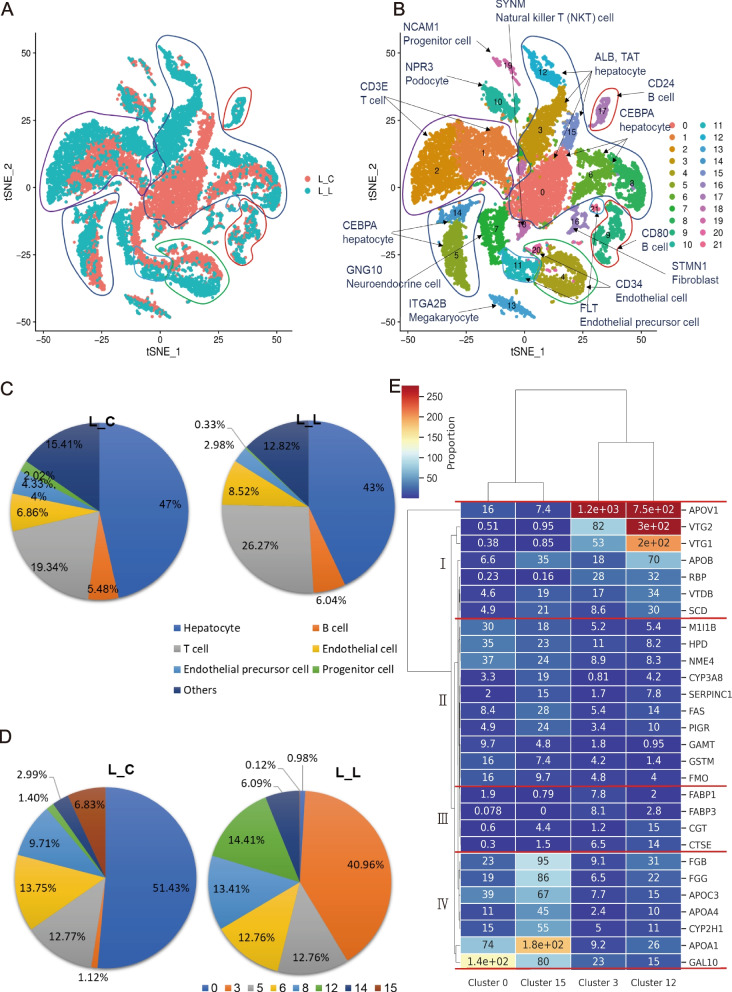
Comparative genomics analysis of liver cells between laying and ceased-laying ducks. **A-B** t-distributed stochastic neighbor embedding (t-SNE) analysis of duck liver cell data. Cells are colored by experimental samples (**A**). Cells are colored by cell type cluster (**B**). Circle of same color represents the cell type, cell types and the marker genes were shown by arrows. **C** The percentage of different cell types in different samples. **D** The percentage of different cell clusters of hepatocytes in two samples. **E** A gene expression heatmap showing the expression of marker genes for cluster 0, 3, 12 and 15 in duck liver data. Rows correspond to the top 10 marker genes in individual clusters; columns are individual cells, ordered by clusters. Red corresponds to high expression level; blue corresponds to low expression level

After quality filtering, a total of 19,096 single cells were captured, 10,583 were from L_C and 8,513 were from L_L (Supplementary Table S3 and Supplementary Figure S2). The 10 main factors were studied by principal components analysis, and the results were visualized in tSNE (Fig. 1A). Cells were clustered into 22 clusters. Normalized expression of the top variable marker genes from 22 clusters are shown in the heatmap (Supplementary Figure S3). Each cluster shows a high specific gene expression pattern. Details were shown in Fig. 1B. *ALB* (albumin), *TAT* (tyrosine aminotransferase isoform X1) and *CEBPA* (CCAAT/enhancer-binding protein alpha) are marker genes of hepatocytes [19, 20], *ALB* and *TAT* were highly expressed in cluster 0,3,12,15, *CEBPA* were highly expressed in cluster 0,6,8,5,14. These faithfully defined cluster 0, 3, 5, 6, 8, 12, 14 and 15 as hepatocytes. Cluster 1 and 2 were marked by *CD3E* (glycoprotein CD3 epsilon chain) [21], indicating its T cells identity. Meanwhile, cluster 9 and 17 showed B cell characteristics, with *CD80* [22] and *CD24* [23] uniquely expressed. Interestingly, we founded a progenitor subpopulation (cluster 19) with extremely high level of *NCAM1* (neural cell adhesion molecule 1 isoform X8) [24]. In addtion, cluster 4 and 20 are defined as endothelial cells by genes like *CD34*. High expression level of *FLT* (vascular endothelial growth factor receptor) [25] indicates cluster 11 and 21 are endothelial precursor cells. According to specific gene expression pattern, cluster 7, 10, 13, 16, 18 were defined as neuroendocrine cell, podocyte, megakaryocyte, fibroblast and natural killer T cell, respectively [26–28]. Hepatocytes of cluster 3 and 12 were unique states in egg-laying ducks, while hepatocytes of cluster 0 and 15 were unique states in ceased-laying duck. This is the main difference between laying and ceased-laying ducks in liver-cell-heterogeneity, while in other cell types, they were almost in homogeneity.

Among all the cell types of two experimental samples, hepatocytes were in dominant (Fig. 1C). In egg-laying duck, hepatocytes accounted for 43.00%, T cells accounted for 26.27%, Endothelial cells accounted for 8.52%, B cells accounted for 6.04%. In ceased-laying duck, hepatocytes accounted for 47.00%, T cells accounted for 19.34%, Endothelial cells accounted for 6.86%, B cells accounted for 5.48%. In general, the percentages of different cell types were similar between two samples. The difference between hepatocytes from two samples were shown in Fig. 1D. Among hepatocytes, cluster 0 and cluster 15 occupied 51.43 and 6.83% in L_C, respectively, while they occupied only 0.98 and 0.12% in L_L. Cluster 3 and cluster 12 occupied 40.96 and 14.41% of hepatocytes in L_L, respectively, while they occupied only 1.12 and 1.40% in L_C. These suggested cluster 0 and cluster 15 were mainly presented in ceased-laying duck, while cluster 3 and cluster 12 were mainly presented in egg-laying duck.

Gene expression heatmap of top 10 marker genes in cluster 0, 3, 12 and 15 was shown in Fig. 1E. For ease of description and understanding, all the genes were divided into 4 parts, I, II III and IV, which were marked in Fig. 1E. From the comparison, cluster 3 and 12 in L_L had higher expression in marker genes of group I and group III, while cluster 0 and 15 in L_C had higher expression in marker genes of group II and group IV. Egg-laying duck highly expressed genes code yolk precursors or transporters, like very-low-density apolipoprotein II (*ApoVLDL-II*, *APOV1*), vitellogenin I (*VTG1*), vitellogenin II (*VTG2*), apolipoprotein B (*APOB*), riboflavin-binding protein (*RBP*/*RTBDN*), vitamin D-binding protein (*VTDB*/*GC*), enzymes in hepatic lipid disposal and in the metabolic utilization of fatty acids, like stearoyl-acyl-CoA desaturase (*SCD*), liver-type fatty acid-binding protein (FABP1) and heart-type fatty acid-binding protein (*FABP3*), and enzymes catalyzing intra oocytic break down of protein components of both vitellogenin and VLDL, like cathepsin E (*CTSE*). Ceased-laying duck highly expressed gallinacin-10 (*GAL10*), fibrinogens (*FGB*, *FGG*), apolipoproteins (*APOA1*, *APOA4*, *APOC3*) and genes related to lipid metabolism, like cytochrome P450 (*CYP3A8*, *CYP2H1*), mid1-interacting protein 1-B (*M1I1B*) and fatty acid synthase (*FAS*/*FASN*), and amino acid metabolism related genes, like 4-hydroxyphenylpyruvate dioxygenase (*HPD*), glutathione S-transferase (*GSTM*), and guanidinoacetate N-methyltransferase (*GAMT*).

### Functional analysis of differentially expressed genes

#### KEGG analysis

**Fig. 2:**
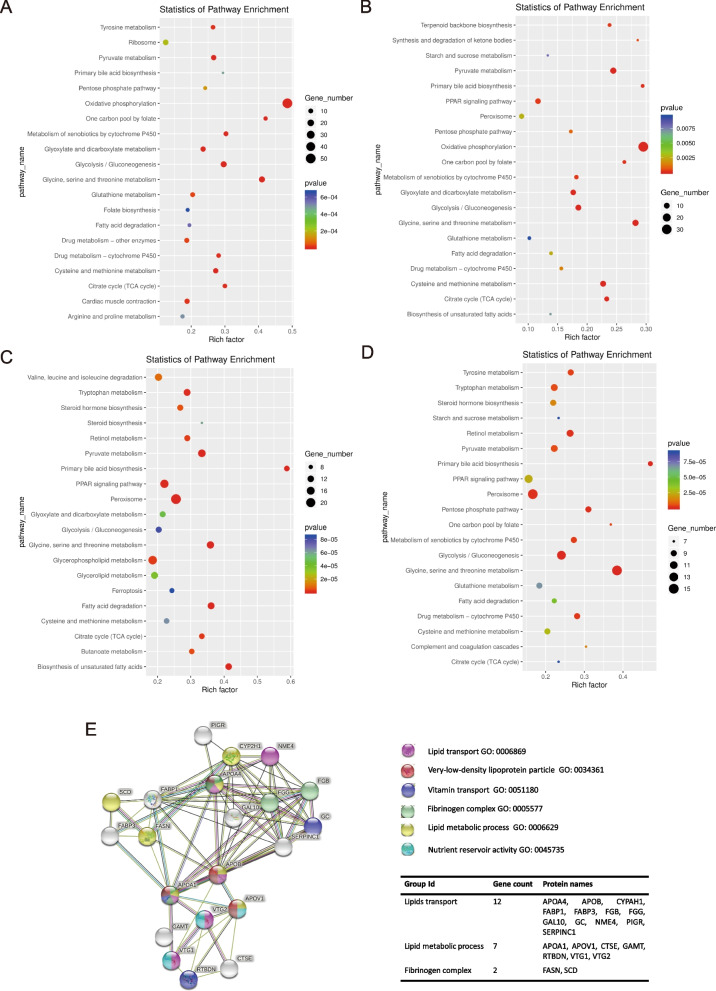
**A-D** The KEGG pathway enrichment of the up-regulated genes in cluster 0 (**A**), cluster 3 (**B**), cluster 12 (**C**) and cluster 15 (**D**). Rich factor represents the ratio of expressed gene number to annotated gene number. Lower number of pvalue equates to higher enrichment. **E** Protein interaction modules of the top 10 marker genes in cluster 0, cluster 3, cluster 12 and cluster 15

The 20 most enriched pathways of cluster 0, 3, 12 and 15 were shown in Fig. 2A-D and Supplementary File 1. All these hepatocytes participated in basic hepatic metabolic pathways, such as primary bile acid biosynthesis, fatty acid degradation, and glycine, serine and threonine metabolism. Cluster 0 and 3 showed a higher function in oxidative phosphorylation than cluster 15 and 12. Genes of Cluster 12, from L_L, enriched in primary bile acid biosynthesis and biosynthesis of unsaturated fatty acids.

#### Protein interaction analysis

The interaction among proteins coding by marker genes of cluster 0, 3, 12, 15 were predicted and shown in Fig. 2E. Basically, all the genes might be divided into three groups, lipids transport, lipid metabolic process and fibrinogen complex (Fig. 2E).

## Discussion

Here, we present a microdroplet-based scRNA-seq technology (*10* × *Genomics*) that enables encapsulation of tens of thousands of single cells within minutes. We analyzed the transcriptomes of ~ 20 k single cells across L_L and L_C. The high-quality transcriptomes, collectively expressing about 17 k genes, provided strong statistical power for unbiased decompositionof these cell populations. Using a conservative statistical threshold, we identified genes that are differentially expressed between cells enabling us to functionally categorize several distinct clusters. To our knowledge, this dataset provides the first cell atlas of duck liver by single-cell transcriptomics profiling. It also provides a fundamental resource to the research of evolution of species.

The relationship between hepatocyte heterogeneity and liver function has been revealed versatilely in human studies. For instance, Ho DW et al. [29] identified a *CD24*^+^/*CD44*^+^-enriched cell subpopulation within the *EPCAM*^+^ cells which had specific signature genes and might indicate a novel stemness-related cell subclone in hepatocellular carcinoma. Zheng H et al. [30] proves that hepatic cancer stem cells (CSCs) at the single-cell level are heterogeneous and different CSC subpopulations are independently associated with hepatocellular carcinoma prognosis. In this sdudy, the effects of hepatocyte heterogeneity on liver function in ceasing laying were focused, which is of scientific significance.

Based on the gene expression, we performed the analysis of cell clustering, cell type analysis and KEGG enrichment analysis. CellMarker is a database that provides a comprehensive and accurate resource of cell markers for various cell types in tissues of human and mouse [31]. CellMarker shows 23 cell types in human and mouse liver. Using the marker genes of liver in CellMarker, we identified 6 cell types, including hepatocytes, T cells, B cells, progenitor cells, endothelial cells and endothelial precursor cells. Other cell types in CellMarker were not identified, such as embryonic cells, stem cells, hematopoietic cells, kupffer cells, dendritic cells, etc. The absence of embryonic cells and stem cells might attribute to the state of the senile ducks. The absence of hematopoietic cells, kupffer cells and denddritic cells might attribute to the loss in cell digestion. In addition, the species differences between birds and mammals might play a role on the bias, e.g., goose and duck have higher liver lipid storage capacity than chicken and mammals [32].

The main function of liver are synthesis, metabolism and decomposition. A large fraction of the corresponding enzymes are mainly expressed in hepatocytes. Our study provided the ocular proof that hepatocytes are dominant in quantitative terms in liver. We also detected a large number of T cells (~ 19–27%) and B cells (~ 5.48–6.04%). A promotion effect of lymph node to liver regeneration has been demonstrated by Fontes et al. [33]. The lymphocytes (T cells and B cells) population in our study might provide a high quality enviroment for liver.

Among all the clusters, we focused on cluster 0, 3, 12 and 15, which showed the main difference between two samples. The cells of these four clusters are hepatocytes. Cluster 3 and 12 specifically belong to egg-laying duck, while cluster 0 and 15 specifically belong to ceased-laying duck. The reference marker gene were used to identify the heterogeneous functions of hepatocytes between ceased-laying duck and egg-laying duck. In egg-laying duck, specific cluster 3 and 12 expressed more yolk materials, like vitellogenins (*VTG1*, *VTG2*) and unsaturated fatty acids (*SCD*), more binding/transport proteins, like *APOV1*, *APOB*, *RBP*, *VTDB*, and more *CTSE*. This is in accordance with the material needs of egg-laying duck, as the yolk formation requires a massive increase in yolk precursor synthesis and package in the liver. As one of the major yolk protein, VTG expressed exclusively in the liver of poultry [34]. SCD is vital to monounsaturated fatty acids (MUFAs) synthesis [9], which is important composition of yolk lipids. APOV1 and APOB are major transport proteins of VLDL-yolk [4]. Richards et al. [35] revealed that hepatic expression of *APOV1* and *APOB* genes increased significantly in broiler breeders after photostimulation at first egg. Riboflavin and vitamin D are important yolk nutrition, here we detected the higher gene expression of their binding proteins, RBP [36] and VTDB [37]. CTSE is also a key enzyme for yolk formation as it is capable of catalyzing intra oocytic break down of protein components of both vitellogenin and VLDL during yolk deposition [38]. For the first, these results show a comprehensive atlas of function genes related to egg laying in a higher resolution of single cells.

In ceased-laying duck, specific cluster 0 and cluster 15 expressed more *FAS*, more other apolipoproteins, like *APOA1*, *APOA4* and *APOC3*, and more blood coagulation related genes, like *FGB*, *FGG* and *SERPINC*. *FAS*, *APOA1* and *APOA4* are involved in tissue-specific fat deposition in chickens [39, 40]. *APOA4* promotes the proliferation of lipoprotein particles and reduces the fat burden in the liver, while not increasing the formation of apolipoprotein B lipoprotein particles, which would increase the possibility of arterial sclerosis [41]. *APOC3* attenuates hydrolysis and clearance of triglyceride-rich lipoproteins [42]. FGB and FGG are polymerize to form an insoluble fibrin matrix, which has a major function in hemostasis, while SERPINC is serine protease inhibitor in plasma that regulates the blood coagulation cascade [43]. These results might explain the higher tissue-specific fat deposition and higher vascular burden in ceased-laying avians.

Our study further proofs that *APOV1* and *APOB* play key roles in egg production, rather than *APOA1* and *APOA4*. It is also the first to detect a correlation between the higher expression of *APOC3*, *FGB*, *FGG* and ceased-laying in duck.

## Conclusions

In conclusion, our study is the first to describe the heterogeneity in the duck liver at different egg-laying period. This single-cell transcriptomic atlas provides a comprehensive genomics resource to study duck in ceasing laying in unprecedented detail. Hepatocytes showed a dominant heterogeneity among all cell types between egg-laying duck and ceased-laying duck. The expression mode of yolk precursor transporters, lipid metabolizing enzymes and fibrinogens were different. *APOV1*, *VTG2*, *VTG1*, *APOB*, *RBP*, *VTDB* and *SCD* might be activated in egg-laying ducks, while *APOA1*, *APOA4*, *APOC3*, *FGB* and *FGG* might be activated in ceased-laying ducks. Our findings provide a solid foundation for future studies on the molecular mechanisms underlying ceasing laying in late phase duck and on strategies for the improvement of egg production.

## Materials and methods

### Animals

Fifty female Shaoxing ducks (1550 ± 92 g, 450 days of age) were randomly selected from one hereditary line at Guowei Poultry Industry Co., Ltd. China, and grown in individual cages under natural temperature and light conditions in October of Zhejiang province. The study is reported in compliance with the ARRIVE guidelines. Feed and water were provided for ad libitum consumption. The ingredients and nutrients of feed were shown in Supplementary Table S4. Successive twelve days of egg production was recorded from 450 days of age. During the twelve days, ducks produced eggs in each day were marked as the egg-laying ducks (L-L), while ducks didn’t produce any egg were marked as the ceased-laying ducks (L-C). One egg-laying duck and one ceased-laying duck were randomly selected. Livers were sampled from egg-laying duck and ceased-laying duck, respectively.

### Preparation of single-cell suspension

The right liver anterior tips were isolated from ducks. The following processing referred to Han et al*.* [16]. Firstly, the samples were quickly transferred into cold PBS (Cat# GNM1514, Genomcell.bio, China) and then minced into ~ 1 mm pieces with scissors. Secondly, they were incubated with 0.25% trypsin–EDTA (Cat# 25,200,056, Gibco, Australia) on ice for 10 min, after washing by cold FBS (Cat# 10,100,147, Gibco, Australia), they were digested by 0.5% collagenase IV (Cat# 17,100,019, Invitrogen, USA) at 37℃ for 40 min with gentle agitation for enzymatic dissociation into single-cell suspensions. Thirdly, the cell suspensions were passed through a 40 μm cell strainer to generate cleaner single-cell suspensions, then, the filtrates were centrifuged for 10 min at 300 × *g* (4℃), and the supernatants were removed. Fourthly, the cells were incubated for 10 min with 1 × RBC lysis buffer at room temperature and centrifuged for 10 min at 300 × *g* (4 °C) to remove the contaminating RBCs. Finally, after removal of the supernatants, the cells were washed and suspended by DPBS (Cat# GNM14190, Genomcell.bio, China) with 1% BSA (Cat# PC0001, Solarbio, China) to the concentration of 7 × 10^5^ cells/cm^2^. The viabilities were assessed using a 0.4% (w/v) trypan blue dye solution (Cat# 15,250,061, Life, USA), and the total numbers of cells were counted using an automated cell counter.

### Library construction and Sequencing

The library construction was referred to Feregrino et al*.* [44]: ① Cell suspensions were loaded on a GemCode Single-Cell Instrument (*10* × *Genomics*, USA) to generate single-cell GEMs (Gel Beads-In-Emulsions). ② Single-cell RNA-Seq libraries were prepared using GemCode Single-Cell 3’Gel Bead and Library Kit (Cat# 1,000,121, *10* × *Genomics*, USA). ③ GEM-RT was performed in a PCR Amplifier (Cat# 1,851,197, Bio-Rad, USA): 55 °C for 2 h, 85 °C for 5 min; held at 4 °C. ④ cDNA products were cleaned up with the SPRI select Reagent Kit (Cat# B23317, Beckman, USA) and subsequently sheared to 200 ~ 300 bp using enzymatic fragmentation system (Cat# 500,295, Covaris, USA). ⑤ Indexed sequencing libraries were constructed using the reagents in the GemCode Single-Cell 3’ Library Kit, following these steps: (1) end repair and A-tailing; (2) adapter ligation; (3) post ligation cleanup with SPRI select; (4) sample index PCR and cleanup. ⑥ The barcode sequencing libraries were quantified by quantitative PCR (Cat# KK4824, KAPA Biosystems Library Quantification Kit for Illumina platforms, USA).

Sequencing libraries were loaded on an Illumina NextSeq 500 with paired-end kits. As shown in Supplementary Figure S4, each sequence contains Illumina P5 adaptor, P5 primer (TruSeq Read 1), Barcode (16 bp), UMI (12 bp), poly (dT) VN, cDNA, P7 primer (TruSeq Read 2), Index (i7 index read) and Illumina P7 adaptor. Reads 1 were used to distinguish different transcripts of different cells. Reads 2 were used to determine the genetic information.

### Data processing

The Cell Ranger Single Cell Software Suite (https://support.10xgenomics.com/single-cell-gene-expression/software/overview/welcome) was applied to perform sample demultiplexing, barcode processing, and single-cell gene counting. The cDNA insert was mapped to an appropriate genome reference (*Anas Platyrhynchos*, ftp://ftp.ncbi.nlm.nih.gov/genomes/all/GCF/003/850/225/GCF_003850225.1_IASCAAS_PekingDuck_PBH1.5/GCF_003850225.1_IASCAAS_PekingDuck_PBH1.5_genomic.fna.gz) using STAR (Spliced Transcripts Alignment to a Reference) [45]. The filter thresholds for mapped data were adapted for each sample, depending on the different library complexities. Such cells were filtered out: cells with an UMI count more than 20,000, cells with a gene count less than 200, and cells with a mitochondrial or ribosomal contribution to UMI count more than 20%. Using the R package Seurat [46], the UMI counts were then Log-normalized.

### Dimensionality reduction and visualization

Significant principal components were determined for each sample as those falling outside of a Marchenko-Pastur distribution. A dimensionality reduction step was carried out following a similar approach to Macosko et al. [47]. K-means [48] clustering on the first 10 principal components were visualized in two-dimensional projection of t-distributed stochastic neighbour embedding projection (tSNE) [49]. Clusters were identified using the Seurat FindClusters function with a resolution parameter of 0.6 for the two physiological stages. The up-regulated genes with *P* < 0.01 and |Log2FC > 0.25| were defined as the marker genes in each cluster compared to all other clusters. The top 10 marker genes in each cluster were selected to construct the heatmap. Cell types were identified by marker genes in CellMarker (http://bio-bigdata.hrbmu.edu.cn/CellMarker/index.jsp) [50] and other published papers.

### KEGG enrichment

KEGG (Kyoto Encyclopedia of Genes and Genomes; http://www.kegg.jp/) [51] is utilized for bioinformatics research in different cell clusters. KOBAS [52] was used to test the statistical enrichment of the up-regulated genes in KEGG pathways.

### Protein interaction prediction

We used STRING (version 11.5) to integrate and rank marker genes associations of cluster 0, 3, 12, 15 by benchmarking them against a common reference set, and presents evidence in a consistent and intuitive web interface [53].

## Supplementary Information

**Additional file 1. **The enriched pathways of gene from cluster 0. The enriched pathways of gene from cluster 15. The enriched pathways of gene from cluster 3. The enriched pathways of gene from cluster 12.

**Additional file 2:** **Table S1.** Statistical results of the sequencing data of liver samples in different laying status.

**Additional file 3:** **Table S2.** Statistical results of quality by Cell Ranger of liver samples in different laying status.

**Additional file 4:** **Table S3. **Statistical table of cell filtration.

**Additional file 5:** **TableS4. **Ingredients and nutrient composition of basal diet.

**Additional file 6:** **Figure S1.** Median number of genes detected per cell as a function of mean reads per cell. L_C: liver of ceased-laying duck; L_L: liver of laying duck.

**Additional file 7:** **Figure S2.** The distribution of basic information of cells in each sample before and after the filter. **A** The distribution of basic information of cells before the filter; **B** The distribution of basic information of cells after the filter; The left image of each group of pictures shows the distribution of gene number of cells in each sample; The middle image of each group of pictures shows the distribution of UMI number of cells in each sample; The right image of each group of pictures shows the distribution of mitochondrial gene expression level of cells in each sample; L_C: liver of ceased-laying duck; L_L: liver of laying duck.

**Additional file 8:** **Figure S3.** Numbers at the top indicate cluster number, with connecting lines indicating the hierarchical relationship between clusters. Representative markers from each cluster are shown on the left.

**Additional file 9:** **FigureS4.** Structure of the library. P5: Illumina P5 adaptor; TruSeq Read 1: P5 primer; 10×Barcode: 16 bp, one bead has one kind of barcode; UMI: 12bp, one transcript matches one kind of UMI; TruSeq Read 2: P7 primer; Index: i7 index read; P7: Illumina P7 adaptor.

## Data Availability

The datasets generated and/or analysed during the current study are available in the NCBI Sequence Read Archive repository, the accession numbers are SRR13402420 and SRR13402419 (https://dataview.ncbi.nlm.nih.gov/object/PRJNA690971).

## Abbreviations

scRNA-seq: Single-cell RNA sequencing
tSNE: T-distributed stochastic neighbor embedding;
UMIs: Unique molecular identifiers
APOV1: Very-low-density apolipoprotein-VLDL II
VTG2: Vitellogenin II
VTG1: Vitellogenin I
APOB: Apolipoprotein B
RBP: Riboflavin-binding protein
VTDB/GC: Vitamin D-binding protein
SCD: Stearoyl-acyl-CoA desaturase
APOA1: Apolipoprotein A1
APOA4: Apolipoprotein A4
APOC3: Apolipoprotein C3
FGB: Fibrinogen B
FGG: Fibrinogen G
VLDL: Very-low-density lipoprotein
PPAR: Peroxisome proliferator activated receptor
LPL: Lipoprotein lipase
DBP: Deep-brain photoreceptor
TSHB: Thyroid-stimulating hormone β
DIO2: Deiodinase 2
OATPs: Organic anion transporting polypeptides
GnRH: Gonadotropin-releasing hormone
LH: Luteinizing hormone
FSH: Follicle-stimulating hormone
*COL1A1*: Type I collagen encoding gene
*ALB*: Albumin
*TAT*: Tyrosine aminotransferase isoform X1
*CEBPA*: CCAAT/enhancer-binding protein alpha
*CD3E*: Glycoprotein CD3 epsilon chain
*CD8B*: Glycoprotein CD8 beta chain
*NCAM1*: Neural cell adhesion molecule 1 isoform X8
*FLT*: Vascular endothelial growth factor receptor
FABP1: Liver-type fatty acid-binding protein
FABP3: Heart-type fatty acid-binding protein
FAS/FASN: Fatty acid synthase
M1I1B: Mid1-interacting protein 1-B
HPD: 4-Hydroxyphenylpyruvate dioxygenase
GSTM: Glutathione S-transferase
GAMT: Guanidinoacetate N-methyltransferase
