# Supplementary material for: Single-cell RNA sequencing revealed the liver heterogeneity between egg-laying duck and ceased-laying duck
Source: BMC Genomics. 2022 Dec 28;23:857. doi: 10.1186/s12864-022-09089-0 (PMC9798604; doi:10.1186/s12864-022-09089-0)
Supplement: Supplementary file 2 — Additional file 2: Table S1. Statistical results of the sequencing data of liver samples in different laying status. [file 12864_2022_9089_MOESM2_ESM.docx]

**Table. S1. Statistical results of the sequencing data of liver samples in different laying status.**

| **Sample** | **Number of Reads** | **Valid Barcodes** | **Sequencing Saturation** | **Q30 Bases in Barcode** | **Q30 Bases in RNA Read** | **Q30 Bases in UMI** |
| --- | --- | --- | --- | --- | --- | --- |
| L_C | 312,569,930 | 96.5% | 65.3% | 95.0% | 93.8% | 92.3% |
| L_L | 284,483,937 | 96.6% | 65.1% | 95.1% | 93.6% | 92.3% |

L_C: liver of ceased-laying duck; L_L: liver of laying duck; Number of Reads：the number of reads obtained from each sample；Valid Barcodes：the ratio of reads with valid barcode to all the reads, each valid barcode represented one cell; Q30 Bases in Barcodes：the percentage of bases in barcode whose mass fraction is greater than or equal to 30；Q30 Bases in RNA Read：the percentage of bases in RNA read whose mass fraction is greater than or equal to 30；Q30 Bases in UMI：the percentage of bases in UMI whose mass fraction is greater than or equal to 30.
