# Supplementary material for: Single-cell RNA sequencing revealed the liver heterogeneity between egg-laying duck and ceased-laying duck
Source: BMC Genomics. 2022 Dec 28;23:857. doi: 10.1186/s12864-022-09089-0 (PMC9798604; doi:10.1186/s12864-022-09089-0)
Supplement: Supplementary file 3 — Additional file 3: Table S2. Statistical results of quality by Cell Ranger of liver samples in different laying status. [file 12864_2022_9089_MOESM3_ESM.docx]

**Table. S2. Statistical results of quality by Cell Ranger of liver samples in different laying status.**

| Sample | ENC | MRC | MGC | RMG | RMCG | RMC (Integenic) | RMC (Intronic) | RMC (Exonic) | RMCT | RMAG | FRC | TGD |
| --- | --- | --- | --- | --- | --- | --- | --- | --- | --- | --- | --- | --- |
| L_C | 10,605 | 29,473 | 538 | 87.6% | 55.9% | 2.8% | 6.0% | 47.1% | 45.7% | 0.5% | 37.4% | 16,627 |
| L_L | 8,620 | 33,002 | 640 | 86.6% | 59.2% | 2.6% | 7.5% | 49.2% | 47.6% | 0.6% | 38.0% | 16,932 |

L_C: liver of ceased-laying duck; L_L: liver of laying duck; ENC: estimated number of cells; MRC: mean reads per cell; MGC: median genes per cell; RMG: reads mapped to genome; RMCG: reads mapped confidently to genome; RMC (Integenic): reads mapped confidently to intergenic regions; RMC (Intronic): reads mapped confidently to intronic regions; RMC (Exonic): reads mapped confidently to exonic regions; RMCT: reads mapped confidently to transcriptome; RMAG: reads mapped antisense to gene; FRC: fraction reads in cells; TGD: total genes detected.
