# Supplementary material for: Single-cell RNA sequencing revealed the liver heterogeneity between egg-laying duck and ceased-laying duck
Source: BMC Genomics. 2022 Dec 28;23:857. doi: 10.1186/s12864-022-09089-0 (PMC9798604; doi:10.1186/s12864-022-09089-0)
Supplement: Supplementary file 4 — Additional file 4: Table S3. Statistical table of cell filtration. [file 12864_2022_9089_MOESM4_ESM.docx]

**Table S3. Statistical table of cell filtration.**

| Sample | before_filter_cell_num | after_filter_cell_num | precent |
| --- | --- | --- | --- |
| L_C | 10605 | 10583 | 99.79% |
| L_L | 8620 | 8513 | 98.76% |

L_C: liver of ceased-laying duck; L_L: liver of laying duck.
