# Supplementary material for: Single-cell RNA sequencing revealed the liver heterogeneity between egg-laying duck and ceased-laying duck
Source: BMC Genomics. 2022 Dec 28;23:857. doi: 10.1186/s12864-022-09089-0 (PMC9798604; doi:10.1186/s12864-022-09089-0)
Supplement: Supplementary file 5 — Additional file 5: TableS4. Ingredients and nutrient composition of basal diet. [file 12864_2022_9089_MOESM5_ESM.docx]

**Table S4. Ingredients and nutrient composition of basal diet.**

| Ingredients | Content(g/kg) |  | Nutrient | Content(g/kg) |
| --- | --- | --- | --- | --- |
| Maize grain | 400 |  | Metabolizable energy | 11.2^b^ |
| Wheat | 290 |  | Crude protein | 16.5 |
| Soybean meal | 120 |  | Total phosphorus | 0.70 |
| Wheat bran | 90 |  | Total calcium | 3.35 |
| Calcium hydrophosphate | 12 |  | Total lysine | 0.79 |
| Stone powder | 80 |  | Total methionine | 0.40 |
| Salt | 3 |  | Ether extract | 29.0 |
| Premix^a^ | 5 |  |  |  |

^a^ Supplied per kg of diet: vitamin A 1,500 U, cholecalciferol 200 U, vitamin E (DL-α-tocopheryl acetate) 10 U, riboflavin 3.5 mg, pantothenic acid 10 mg, niacin 30 mg, cobalamin 10 μg, choline chloride 1,000 mg, biotin 0.15 mg, folic acid 0.5 mg, thiamine 1.5 mg, pyridoxine 3.0 mg, Fe 80 mg, Zn 40 mg, Mn 60 mg, I 0.18 mg, Cu 8 mg, Se 0.3 mg; ^b^ Unit: MJ/kg
