# Supplementary material for: Single-cell RNA sequencing revealed the liver heterogeneity between egg-laying duck and ceased-laying duck
Source: BMC Genomics. 2022 Dec 28;23:857. doi: 10.1186/s12864-022-09089-0 (PMC9798604; doi:10.1186/s12864-022-09089-0)
Supplement: Supplementary file 6 — Additional file 6: Figure S1. Median number of genes detected per cell as a function of mean reads per cell. L_C: liver of ceased-laying duck; L_L: liver of laying duck. [file 12864_2022_9089_MOESM6_ESM.docx]

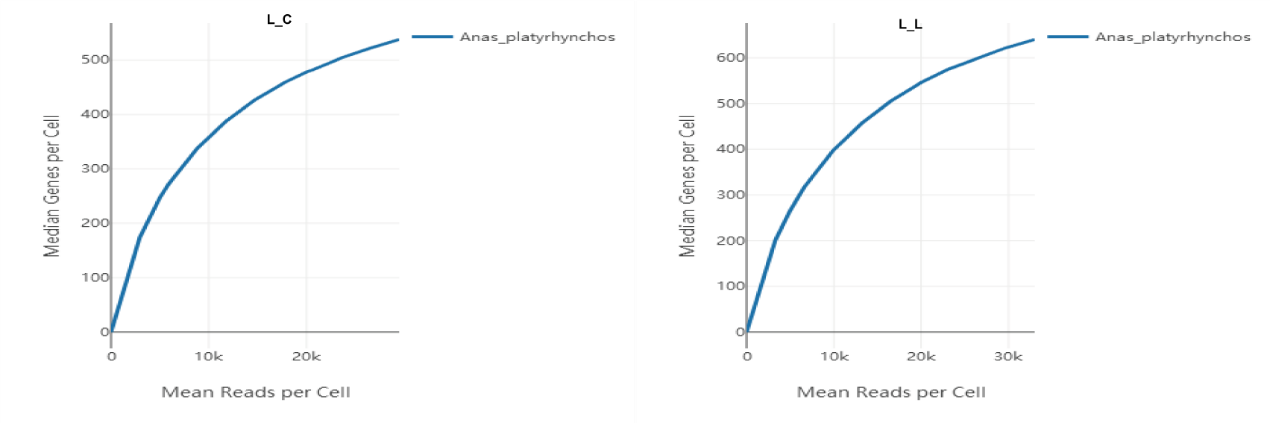


**Figure S1.** Median number of genes detected per cell as a function of mean reads per cell. L_C: liver of ceased-laying duck; L_L: liver of laying duck.
