# Supplementary material for: Single-cell RNA sequencing revealed the liver heterogeneity between egg-laying duck and ceased-laying duck
Source: BMC Genomics. 2022 Dec 28;23:857. doi: 10.1186/s12864-022-09089-0 (PMC9798604; doi:10.1186/s12864-022-09089-0)
Supplement: Supplementary file 7 — Additional file 7: Figure S2. The distribution of basic information of cells in each sample before and after the filter. A The distribution of basic information of cells before the filter; B The distribution of basic information of cells after the filter; The left image of each group of pictures shows the distribution of gene number of cells in each sample; The middle image of each group of pictures shows the distribution of UMI number of cells in each sample; The right image of each group of pictures shows the distribution of mitochondrial gene expression level of cells in each sample; L_C: liver of ceased-laying duck; L_L: liver of laying duck. [file 12864_2022_9089_MOESM7_ESM.docx]

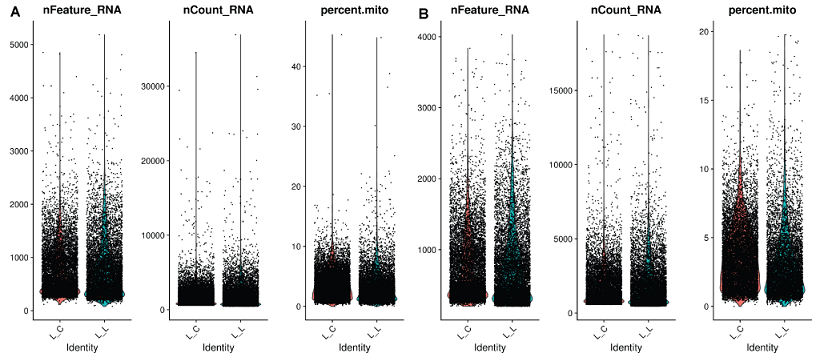


**Figure S2.** The distribution of basic information of cells in each sample before and after the filter. **A** The distribution of basic information of cells before the filter; **B** The distribution of basic information of cells after the filter; The left image of each group of pictures shows the distribution of gene number of cells in each sample; The middle image of each group of pictures shows the distribution of UMI number of cells in each sample; The right image of each group of pictures shows the distribution of mitochondrial gene expression level of cells in each sample; L_C: liver of ceased-laying duck; L_L: liver of laying duck.
