# Supplementary material for: Single-cell RNA sequencing revealed the liver heterogeneity between egg-laying duck and ceased-laying duck
Source: BMC Genomics. 2022 Dec 28;23:857. doi: 10.1186/s12864-022-09089-0 (PMC9798604; doi:10.1186/s12864-022-09089-0)
Supplement: Supplementary file 8 — Additional file 8: Figure S3. Numbers at the top indicate cluster number, with connecting lines indicating the hierarchical relationship between clusters. Representative markers from each cluster are shown on the left. [file 12864_2022_9089_MOESM8_ESM.docx]

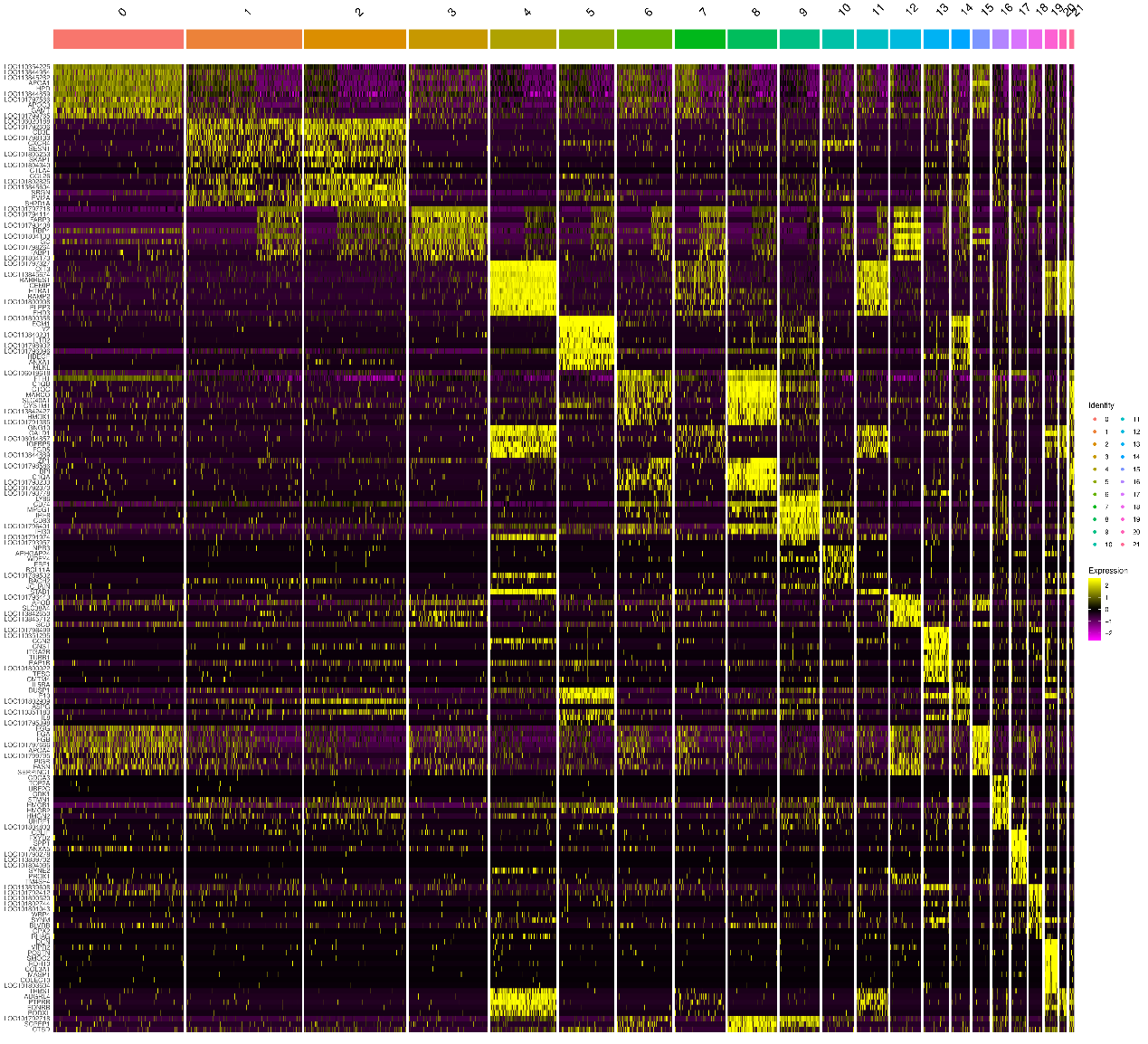


**Figure S3.** Numbers at the top indicate cluster number, with connecting lines indicating the hierarchical relationship between clusters. Representative markers from each cluster are shown on the left.
