# Supplementary material for: Single-cell RNA sequencing revealed the liver heterogeneity between egg-laying duck and ceased-laying duck
Source: BMC Genomics. 2022 Dec 28;23:857. doi: 10.1186/s12864-022-09089-0 (PMC9798604; doi:10.1186/s12864-022-09089-0)
Supplement: Supplementary file 9 — Additional file 9: FigureS4. Structure of the library. P5: Illumina P5 adaptor; TruSeq Read 1: P5 primer; 10×Barcode: 16 bp, one bead has one kind of barcode; UMI: 12bp, one transcript matches one kind of UMI; TruSeq Read 2: P7 primer; Index: i7 index read; P7: Illumina P7 adaptor. [file 12864_2022_9089_MOESM9_ESM.docx]

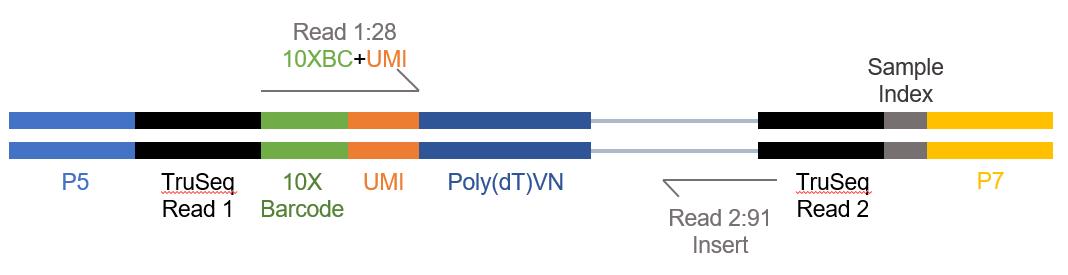


**Figure S4.** Structure of the library. P5: Illumina P5 adaptor; TruSeq Read 1: P5 primer; 10×Barcode: 16 bp, one bead has one kind of barcode; UMI: 12bp, one transcript matches one kind of UMI; TruSeq Read 2: P7 primer; Index: i7 index read; P7: Illumina P7 adaptor.
